# Efficacy and Safety of ^225^Ac-PSMA-617-Targeted Alpha Therapy in Metastatic Castration-Resistant Prostate Cancer: A Systematic Review and Meta-Analysis

**DOI:** 10.3389/fonc.2022.796657

**Published:** 2022-02-03

**Authors:** Jiao Ma, Lanying Li, Taiping Liao, Weidong Gong, Chunyin Zhang

**Affiliations:** ^1^ Department of Nuclear Medicine, The Affiliated Hospital of Southwest Medical University, Luzhou, China; ^2^ Nuclear Medicine and Molecular Imaging Key Laboratory of Sichuan Province, Luzhou, China; ^3^ Academician (expert) Workstation of Sichuan Province, Luzhou, China

**Keywords:** ^225^Ac-PSMA-617, α nuclide therapy, metastatic castration-resistant prostate cancer, meta-analysis, systematic review

## Abstract

**Objective:**

To conduct a meta-analysis of the efficacy and safety of ^225^Ac-PSMA-617 in the treatment of metastatic castration-resistant prostate cancer based on existing clinical evidence.

**Methods:**

Search for retrospective studies about ^225^Ac-PSMA-617 in the treatment of metastatic castration-resistant prostate cancer from establishment to July 2021 in PubMed and EMBASE. The primary endpoint was ^225^Ac-PSMA-617 biochemical response evaluation criteria after treatment [any prostate specific antigen (PSA) decrease and PSA decrease >50% from baseline] to evaluate the treatment effect. Secondary endpoints included assessment of overall survival (OS), progression-free survival (PFS), molecular response, and toxicity for all studies. Two researchers conducted literature screening, data extraction and quality evaluation according to the inclusion and exclusion criteria. Use stata16.0 software for analysis, fixed-effects model for data merging and forest plots for display.

**Results:**

A total of 6 retrospective studies, namely, 201 patients, were included in the final analysis. The pooled proportions of patients with decreased PSA and PSA decreased by more than 50% were 87.0% (95% confidence interval, 0.820 to 0.920) and 66.1% (95% confidence interval, 0.596 to 0.726), respectively. The pooled proportions of OS and PFS were 12.5 months (95%CI: 6.2–18.8 months) and 9.1 months (95%CI: 2.6–15.7 months). The patients showing molecular responses were 54% (95% confidence interval: 25–84%). In all studies, the most common side effect of ^225^Ac-PSMA-617 TAT was xerostomia, with any degree of xerostomia occurring in 77.1% (155 out of 201), and grade III only accounted for 3.0%. The second was 30.3% (61 out of 201) anemia of any degree, and grade III accounts for 7.5% (15 out of 201). Grade III leukopenia and thrombocytopenia were 4.5% (9 out of 201) and 5.5% (11 out of 201), respectively. Only 6 (3.0%) of 201 patients had Grade III nephrotoxicity.

**Conclusion:**

^225^Ac-PSMA-617 is an effective and safe treatment option for mCRPC patients, and the toxicity caused by it is relatively low. However, future randomized controlled trials and prospective trials are required in the future to judge the therapeutic effects and survival benefits compared with existing clinical treatments.

**Systematic Review Registration:**

PROSPERO: CRD42021281967.

## Introduction

Prostate cancer is one of the most common malignant tumors in men around the world. According to the latest report of global cancer statistics in 2020, the incidence and mortality rates of prostate cancer rank the 2nd and 5th among malignant tumors in men around the world (1). At present, the main treatment methods for prostate cancer include radical surgical resection, radiotherapy, chemotherapy, local radiotherapy, androgen deprivation therapy, targeted therapy, and immunotherapy. As the condition of the patient progresses, the efficacy of these therapies will gradually decrease or even be completely ineffective (2). For advanced prostate cancer, androgen deprivation therapy has an effective effect. In the stage of metastatic emasculation-sensitive prostate cancer (mCSPC), combination therapy can improve survival rate than ADT alone (3). But there is still a lack of consensus on the best treatment options. Studies have shown that compared with docetaxel, androgen receptor axis targeting (ARAT) drugs may better improve the outcome of OS. However, the best treatment option remains to be determined (4). Most patients will become castration resistant after a period of 1 to 2 years of androgen sensitivity. The emergence of a state of castration resistance will lead to rapid progress of the disease, accelerate the metastasis of prostate cancer, and eventually progress to metastatic castration-resistant prostate cancer, leading to the ineffectiveness of chemotherapy and castration treatment. This is also the main cause of death in prostate cancer patients (5). Drugs such as abiraterone acetate, enzalutamide, carbachol, and apalutamide have good treatments for patients with mCRPC.In addition, olaparib and rucaparib can be used to treat mCRPC with BRCA gene mutations. Pembrolizumab is the first PD-1 inhibitor approved to treat prostate cancer. However, these drugs have unclear resistance mechanisms, and most patients will develop congenital or acquired resistance after treatment. The first α nuclide radiopharmaceutical approved by the US FDA for clinical treatment, ^223^Ra-dichloride, is suitable for the treatment of patients with CRPC with symptomatic bone metastases and no known visceral metastases. In order to improve the clinical symptoms, overall survival (OS) and quality of life of patients, new drugs are being studied and are developing rapidly. However, the demand for effective treatments for mCRPC has not yet been met. We still lack effective treatments to treat patients at this stage of the disease. Therefore, there is an urgent need to find a new method with high efficiency, safety and low recurrence rate to treat mCRPC.

In recent years, radionuclide-labeled prostate-specific membrane antigen ligands have been used in the diagnosis and treatment of prostate cancer, and have achieved promising results. Prostate-specific membrane antigen is a membrane glycoprotein that is overexpressed on prostate cancer cells. Compared with normal prostate tissue, its expression level in prostate cancer tissue has increased by about 100–1,000 times. There is a direct correlation between androgen independence, metastasis, and disease progression, making PSMA an ideal target for diagnosis and treatment. ^177^Lu-PSMA-617, which emits beta rays, has shown good effectiveness, safety, and easy availability for mCRPC, and has high clinical value and application prospects (6, 7). However, most patients still tolerate ^177^Lu treatment or their condition continues to progress after ^177^Lu and this treatment is contraindicated for patients with diffuse red bone marrow infiltration (8).

The half-life of ^225^Ac is 10.0 d and the decay can produce 6 daughter nuclides, and each decay process releases 4 alpha particles, 2 beta particles and 2 gamma photons (9). Compared with ^177^Lu, ^225^Ac ray has higher energy, shorter range, and stronger killing effect on tumor cells. In addition, ^225^Ac-PSMA-617 also has the advantage of targeting any metastatic tissue, and it has a good application prospect for small tumors, scattered cancers and micrometastasis (10). At present, ^225^Ac-PSMA-617 for mCRPC has been gradually undergoing clinical trials in multiple centers to evaluate its efficacy and safety. However, due to the small sample size, population heterogeneity and different results, there are few systematic reviews or meta-analysis studies on the efficacy and safety of ^225^Ac-PSMA-617 targeted therapy for mCRPC in the published literature. This study will meta-analyze the current published clinical studies on the treatment of mCRPC with ^225^Ac-PSMA-617, in the hopes of providing evidence-based medicine for the efficacy and safety of ^225^Ac-PSMA-617 in the treatment of mCRPC.

## Materials and Methods

This systematic review followed the Preferred Reporting Items for Systematic Reviews and Meta-Analysis (PRISMA) guidelines (11). The registration number on PROSPERO is: CRD42021281967.

### Search Strategy

Articles were searched in PubMed and Embase for articles published until July 2021 about ^225^Ac-PSMA-617 in the treatment of metastatic castration-resistant prostate cancer. The search keywords were as follows: [prostate* neoplasm* (Mesh) OR prostate cancer] AND [Actinium-225 (Mesh) OR 225Ac OR 225Actinium OR Ac-225]. All retrospective studies were searched and appropriate data were included for analysis. If the article meets the research criteria, the full text will be searched. If there were duplications (patient data from the same trial or institution), only the most complete, up-to-date and relevant studies were selected.

### Study Selection and Quality Assessment

We only selected studies that meet the following criteria: Participants (P) were no less than 10 people who had been diagnosed as mCRPC through ^68^Ga-PSMA-11 PET/CT. Interventions (I) were completed at least 1 cycle of ^225^Ac-PSMA-617 treatment; If data came from the same study group, the study with the highest number of patients will be included. The main outcome endpoint (O) was any decrease in PSA and Greater than 50% PSA decline. The type of study (S) included in the article was retrospective research. Exclusion criteria include: mCRPC patients suffering from severe leukopenia, low platelets, renal failure, and those who cannot tolerate ^225^Ac-PSMA-617 treatment in the terminal stage of cancer; Patients with hormone-sensitive prostate cancer receive ^225^Ac-PSMA-617 targeted radiotherapy; Repeated studies, meta-analysis, reviews, case reports, brief communications, abstracts, letters to the editor. The Newcastle–Ottawa Scale (NOS) scale was used to evaluate the literature methodological quality of the selected studies. The quality scale was divided into three categories: selectivity (1 to 4 points), comparability (1 to 2 points), and results (1 to 3 points). According to the scores from these three aspects, the quality of the literatures with NOS ≥6 points was better (**Table 1**).

**Table 1:** Quality assessment of the included studies based on the Newcastle–Ottawa Scale.

NO.	Author and year	Selection	Comparability	Outcome	Score
1	Kratochwil et al. (12)	3	1	3	7
2	Sathekge et al. (13)	3	1	3	7
3	van der Doelen et al. (14)	3	1	3	7
4	Satapathy et al. (15)	3	1	2	6
5	Feuerecker et al. (16)	2	1	3	6
6	Sen et al. (17)	3	1	3	7

### Data Extraction

Two researchers independently conducted a literature search and extracted data. If there was a dispute, this was discussed and resolved with a third person. The basic research data extracted included: author name, publication year, patient demographics, Gleason score, Eastern Cancer Cooperation Group performance score, and baseline level (**Table 2**). Observation indicators included tumor markers (PSA), number of ^225^Ac-PSMA-617 treatment cycles, follow-up interval, dose and drug activity, and primary outcome endpoint was biochemical response. Secondary outcome endpoints included overall survival (OS), progression-free survival (PFS), molecular reactions, and toxicity (**Tables 3**–**5**). The biochemical response was evaluated according to the criteria defined by the Prostate Cancer Clinical Trials Working Group 3 (PCWG3) (18). Patients with greater than 50% PSA decline from baseline were defined as a biochemically significant response, and any decrease in PSA level was recorded. The molecular response was scanned on ^68^Ga-PSMA PET/CT, evaluated according to adjusted PERSIST 1.0 (19), and the proportion of patients with complete response (CR) and partial response (PR) was combined as the molecular response rate. PFS was defined as the time from the first dose of ^225^Ac-PSMA-617 to the first evidence of progression or death or the end of the study period; OS was defined as the time from the first dose of ^225^Ac-PSMA-617 to death from any cause. Toxicity was defined according to the Common Terminology Standard for Adverse Events Version 5.0 (CTCAE 5.0) (20).

**Table 2:** Basic characteristics of the included studies.

Author and year		Patients (n)	Age (yr) (Median, Range)	Baseline PSA (ng/ml) (Median)			GS	ECOG
Kratochwil et al. (12)		40	70	169			NR	0–1 (80%)
								≥2 (20%)
Sathekge et al. (13)		73	69 (45–85)	57.2			8 (6–10)	0–1 (82%)
								2–3 (18%)
van der Doelen et al. (14)		13	71 (64–77)	878 (203–1611)			≥8 6 (46.2%)	0 (23.1%)
								1–2 (76.9%)
Satapathy et al. (15)		11	68 (57–81)	158 (35–840)			8 (7–9)	0–1 (64%)
								2 (36%)
Feuerecker et al. (16)		26	72.5 (63–75.8)	331 (142–682)			8 (7–9)	1 (0-1)
Sen et al. (17)		38	68 (53–84)	NR			7≤ (10.5%)	≤2 (100%)
							≥8 (89.5%)	
Author and year	Doce-taxel	Enzaluta-mide	Previous treatment (%)	Cabazit-axel	^223^Ra-Cl_3_	Olaparib	^177^Lu-PSMA	Site of metastases at baseline
			Abirateronea-cetate					
Kratochwil et al. (12)	70%	60%	85%	17.5%	22.5%	NR	NR	Skeletal:97.5%;liver:22; lung:22.5%;brain:5%; others: 7.5%
Sathekge et al. (13)	NR	1%	1%	NR	NR	NR	14%	skeletal:90%;Isolated lymph node: 10%; liver:5%;lung:3%;brain:1%
van der Doelen et al. (14)	100%	76.9%	84.6%	61.5%	30.8%	63.6%	15.4%	skeletal:100%;Lymph node: 77%;visceral: 62%
Satapathy et al. (15)	91%	36%	64%	27%	NR	NR	46%	skeletal: 100%; Lymph node: 82%;
Feuerecker et al. (16)	96%	85%	88%	54%	23%	NR	100%	skeletal:100%;Lymph node:88%;liver:19%; lung:23%; others: 19%
Sen et al. (17)	100%	34%	63%	10.50%	5.20%	10.50%	23.6%	skeletal:47%;Lymph node:34%;lung:11%; liver:8%

NR, not reported; PSA, prostate‐specific antigen; ECOG, Eastern Cooperative Oncology Group; GS, Gleason score.

**Table 3:** The treatment characteristics of the included studies.

Author and year	Patients Analyzed for PSA Decline (n)	Dose	Cycles of Therapy (Median, Range)	Follow-Up (wk)	Any PSA Decline (%)	PSA Decline >50%
Kratochwil et al. (12)	38	100 KBq/kgBW	1–3	8	33/38 (87)	24/38 (63)
Sathekge et al. (13)	73	4–8 MBq/cycle	3 (1–8)	8	60/73 (83)	51/73 (70)
van der Doelen et al. (14)	13	6–8 MBq/cycle	3 (1–4)	8	NR	9/13 (69)
Satapathy et al. (15)	11	100 KBq/kgBW	2 (1–4)	8–12	NR	5/11 (46)
Feuerecker et al. (16)	26	9 MBq/cycle	2 (1–6)	8	23/26 (88)	17/26 (65)
Sen et al. (17)	38	100 KBq/kgBW	2 (2–5)	8	33/38 (87)	25/38 (66)

NR, not reported; BW, body weight; PSA, prostate-specific antigen.

**Table 4:** The treatment characteristics of the included studies.

Author and Year	Patients (n)	Molecular Response n/N (%)	OS (Months) (Median, Range)	PFS (Months) (Median, Range)	Treatment Related Deaths, n/N (%)
Kratochwil et al. (12)	40	NR	>12.0 (NR)	7.0 (NR)	NR
Sathekge et al. (13)	73	21/73 (29)	18 (16.2–19.9)	15.2 (13.1–17.4)	NR
van der Doelen et al. (14)	13	6/7 (86)	8.5 (NR)	5.5 (NR)	NR
Satapathy et al. (15)	11	NR	NR	NR	3/11 (27)
Feuerecker et al. (16)	26	NR	7 (4.5–12.1)	3.5 (1.8–11.2)	NR
Sen et al. (17)	38	17/38 (45)	12 (9.1–14.9)	8 (5.3–10.6)	NR

NR, not reported; OS, overall survival; PFS, progression-free survival.

**Table 5:** Treatment-related toxicity of the included studies.

Author and Year	Patients (n)	Hematological Toxicity n/N (%)	Nephrotoxicity n/N (%)	Xerostomia, n/N (%)	Other Manifestation
		Any grade Grade ≥3	Any grade Grade ≥3	Any grade Grade ≥3	
Kratochwil et al. (12)	40	NR	NR	19/40 (47.5) NR	NR
Sathekge et al. (13)	73	① 27/73 (37) 5/73 (7)	23/73 (32) 5/73 (7)	62/73 (85) 0/73 (0)	Grade1/2 nause
		② 9/73 (12) 2/73 (3)			15/73 (21)
		③ 7/73 (10) 1/73 (1)			Anorexia 23/73
					(32),
					Constipation
					19/73 (26),
					Fatigue 37/73 (51),
					Weightloss 28/73 (38),
					Hypoalbuminemia
					14/73 (19),
					Dysuria 13/73 (18),
					xerophthalmia 4/73 (6)
Van der Doelen et al. (14)	13	① 0/13 (0) /	0/13 (0) /	3/13 (100) 0/13 (0)	swallowing, speech, dysgeusia 13/13 (100)
		② 0/13 (0) /			
		③ 0/13 (0) /			
Satapathy et al. (15)	11	① 8/11 (73) 1/11 (9)	1/11 (9) 1/11 (9)	8/11 (73) 1/11 (9)	Grade1/2 nausea 2/11 (18),
		② 5/11 (46) 0/11 (0)			Constipation 2/11 (18),
		③ 5/11 (46) 2/11 (18)			Fatigue 3/11 (27),
					Weightloss 2/11 (18),
					Anorexia 3/11 (27)
Feuerecker et al. (16)	26	①15/26 (58) 9/26 (35)	5/26 (19) 0/26(0)	26/26 (100) 0/26(0)	Grade1 fatigue
		②13/26 (50) 7/26 (27)			12/26 (36),
		③14/26 (54) 5/26 (19)			Weightloss 3/26 (12),
					anorexia 8/26 (31)
Sen et al. (17)	38	①11/20 0/20	NR	37/38 (97) 5/38()	Weightloss 21/38 (55),
		②3/38 0/38			Grade IV
		③4/38 3/38			Hearing loss 2/38 (),
					GradeI/2 nausea 9/38,

① Anemia; ② leucopenia; ③ Thrombocytopenia; NR, not reported.

### Statistical Analyses

Stata16.0 was used for meta-analysis. The main endpoint was to evaluate the treatment effect through the biochemical response evaluation standard after ^225^Ac-PSMA-617 treatment (any decrease in PSA and greater than 50% PSA decline). Secondary endpoints included OS, PFS, molecular reactions, and toxicity, and drawing forest maps for analysis. I^2^ statistic was used for heterogeneity test. If there was no significant heterogeneity among studies (I^2^ ≤50%, P <0.10), a fixed effect model was used to merge data. If there was significant heterogeneity among the studies (I^2^ >50%, P ≥0.10), the random effect model was used to merge the data. The funnel chart and Egger test were used to evaluate the publication bias of the biochemical response after ^225^Ac-PSMA-617 treatment, and P ≤0.05 was considered statistically significant.

## Results

### A Systematic Review of Literature

**Figure 1:**
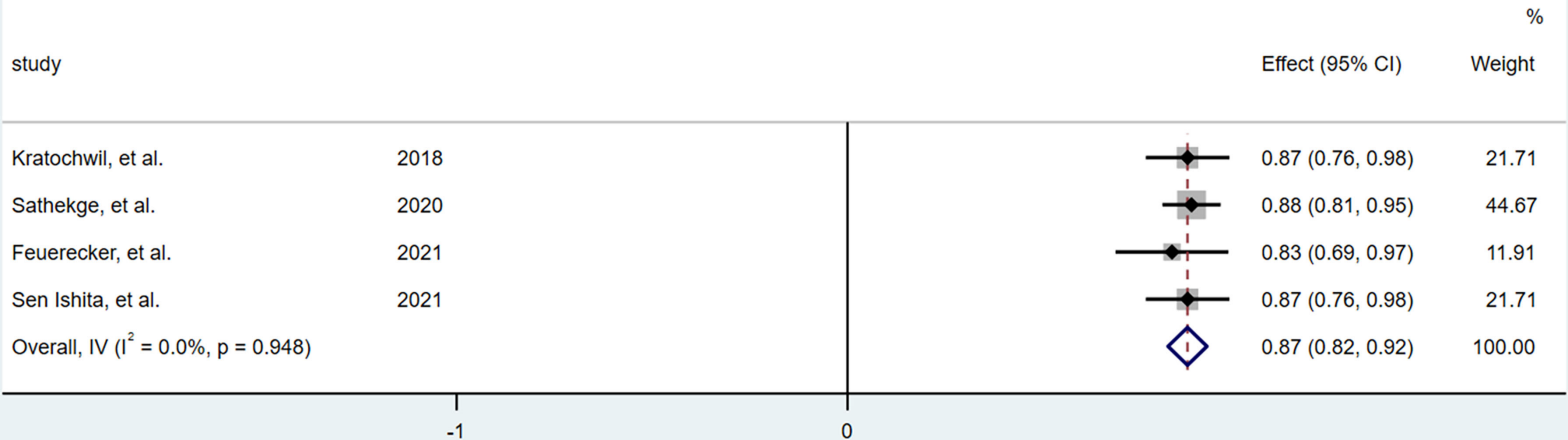
Flowchart of literature screening.

According to the prescribed search strategy, a total of 176 related articles were first checked out. A total of 64 duplicate articles were excluded. A total of 99 articles were excluded by reading titles and abstracts, namely, 43 reviews, 22 preclinical studies, 9 radiochemistry, 8 case reports and brief communications, and 8 dosimetry and imaging related articles, 5 other alpha nuclide therapies and not related to ^225^Ac-PSMA treatment, 2 meta-analysis, 1 ^225^Ac-PSMA resistance gene sequencing, and 1 ^225^Ac-PSMA-I&T treatment. After further reading the full text, and according to the inclusion and exclusion criteria designed in this study, 7 articles were excluded. An article by Sathekge et al. (21) reported about ^225^Ac-PSMA-617 in chemotherapy-naive patients. Two articles by Kratochwil et al. (22, 23), first discussed about only 2 patients, and the other on a dose escalation study of ^225^Ac-PSMA-617. A prospective study was also done by Yadav et al. (24). Three articles reported using ^225^Ac-PSMA-617/^177^Lu-PSMA-617 tandem treatment (25–27). Finally, a total of 6 articles were included (12–17), as shown in **Figure 1**.

### Any PSA Decline

**Figure 2:**
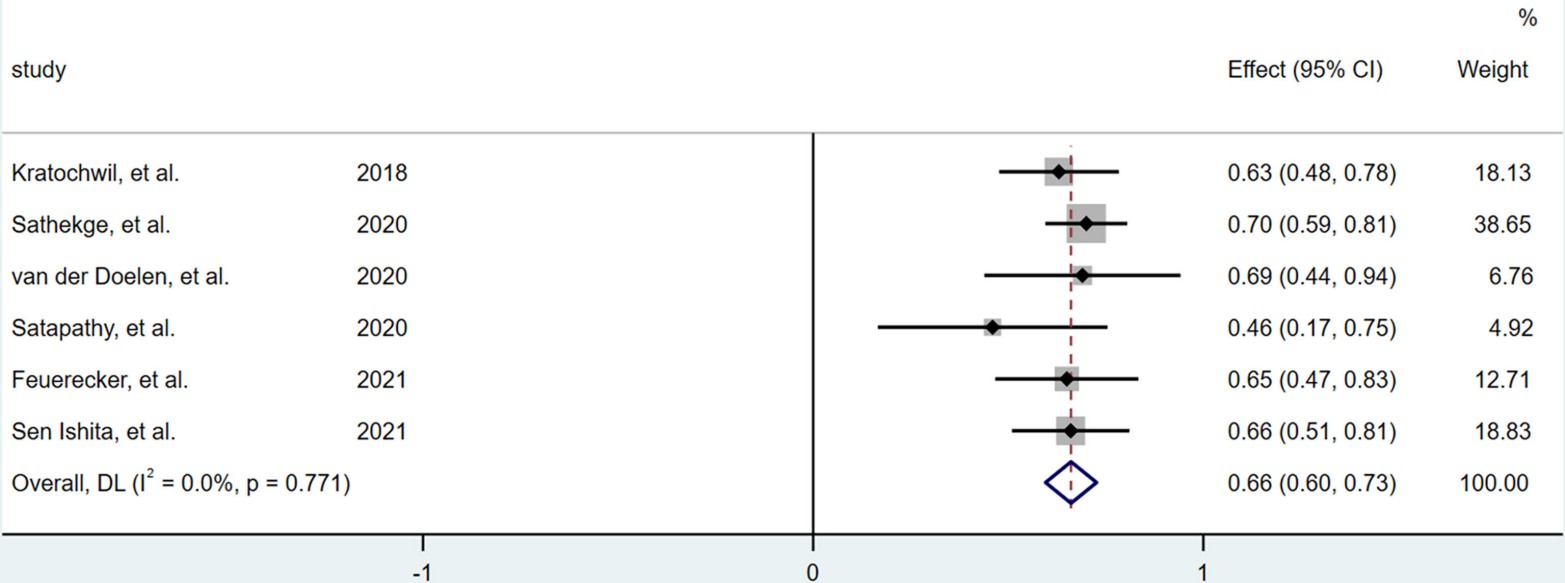
Forest plot for any PSA decline after treatment.

A total of 4 articles (12, 13, 16, 17) were included in the analysis. Among 201 patients, 175 patients were evaluated with a decline of PSA level, and 149 patients had any decline of PSA. The result of heterogeneity analysis showed that there was no significant heterogeneity (I^2^ = 0.0%, P = 0.948), so the fixed effect model was used to merge the PSA reduction rate. The result of meta-analysis showed that the pooled rate of PSA decline after treatment with ^225^Ac-PSMA-617 was 0.870 (95%CI: 0.820–0.920), as shown in **Figure 2**.

### Greater Than 50% PSA Decline

**Figure 3:**
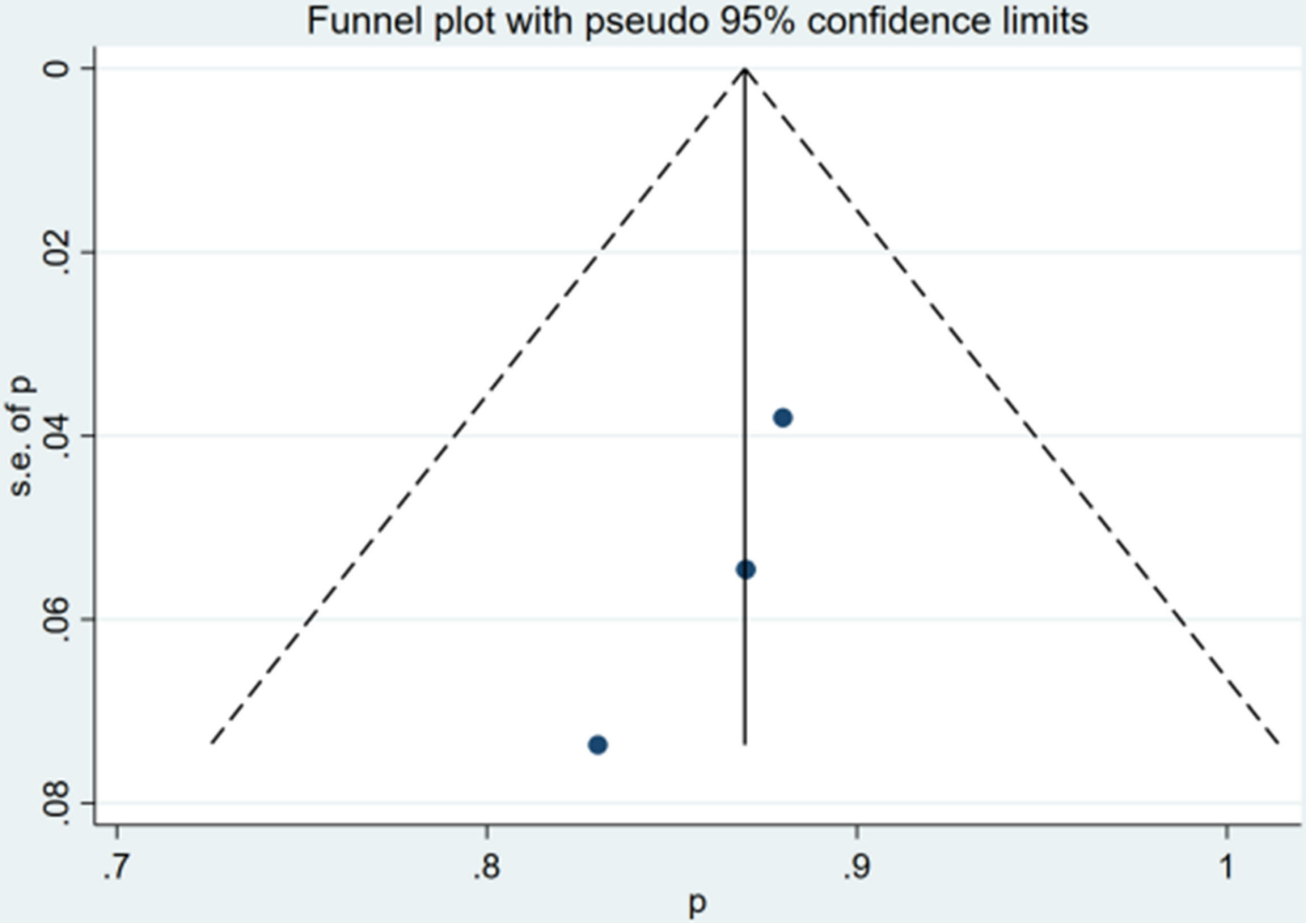
Forest plot for >50% PSA decline after treatment.

A total of 6 articles (12–17) were included in the analysis. Among 201 patients, 199 patients were evaluated, and 131 patients had PSA >50% decline. The results of heterogeneity analysis showed that there was no significant heterogeneity (I^2^ = 0.0%, P = 0.771), so the fixed effect model was used to merge the PSA reduction rate of greater than 50%.The forest plot indicated (**Figure 3**) that the pooled rate of greater than 50% PSA decline was 0.661 (95%CI: 0.596–0.726).

### Survival

OS and PFS were reported in 5 studies (12–14, 16, 17). But in only 3 studies,137 patients, the median of OS and PFS and 95% confidence interval were reported (13, 16, 17). The pooled estimates of median OS and PFS were 12.5 months (95%CI: 6.2–18.8 months) and 9.1 months (95%CI: 2.6–15.7 months).

### Molecular Response

The molecular response was evaluated according to the adjusted PERSIST 1.0, and the complete reaction (CR) and partial reaction (PP) were combined as molecular response. There were 3 studies that met the evaluation requirements (13, 14, 17), namely, 124 patients, and the pooled proportion of patients with molecular response was 54% (95%CI: 25–84%).

### Toxicity

According to the Common Terminology Standard for Adverse Events Version 5.0 (CTCAE 5.0), the toxicity of ^225^Ac-PSMA-617 TAT was analyzed in 6 studies. Xerostomia was the most common side effect. Xerostomia of any degree accounted for 77.1% (155 out of 201 people), and only 6 people had grade III xerostomia, occurring in 3.0%. Then anemia was 30.3% (61 out of 201 people), and grade III anemia was 7.5% (15 out of 201 people). Leukopenia and thrombocytopenia of any degree were 14.9 (30 out of 201); grade III leukopenia and thrombocytopenia were 4.5% (9 out of 201) and 5.5% (11 out of 201). Only 6 (3.0%) of 201 patients had Grade III nephrotoxicity. Other adverse reactions included weight loss 26.9% (54 out of 201), fatigue 25.9% (52 out of 201), anorexia 16.9% (34 out of 201), nausea 12.9% (26 out of 201), and constipation 10.4% (21 out of 201). In addition, in the study of Sathekge (13), 4 patients had symptoms of xerophthalmia, and the study of Sen (17) reported 2 patients with hearing loss. Among the evaluable patients, treatment-related deaths were reported in only one study (15), and 3 of 11 patients had treatment-related deaths.

### Risk of Bias

**Figure 4:**
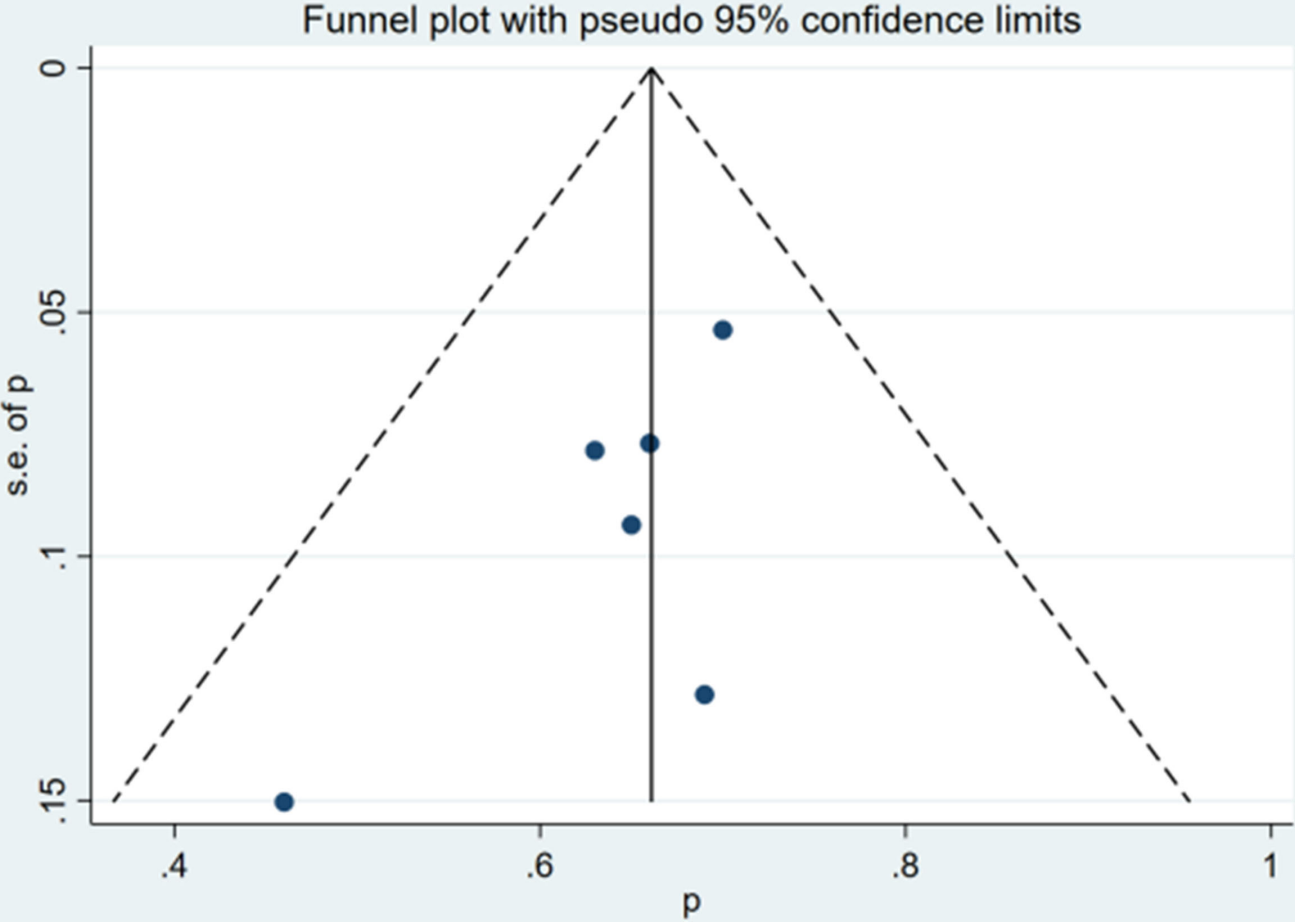
Funnel plot for any PSA decline after treatment.

**Figure 5:**
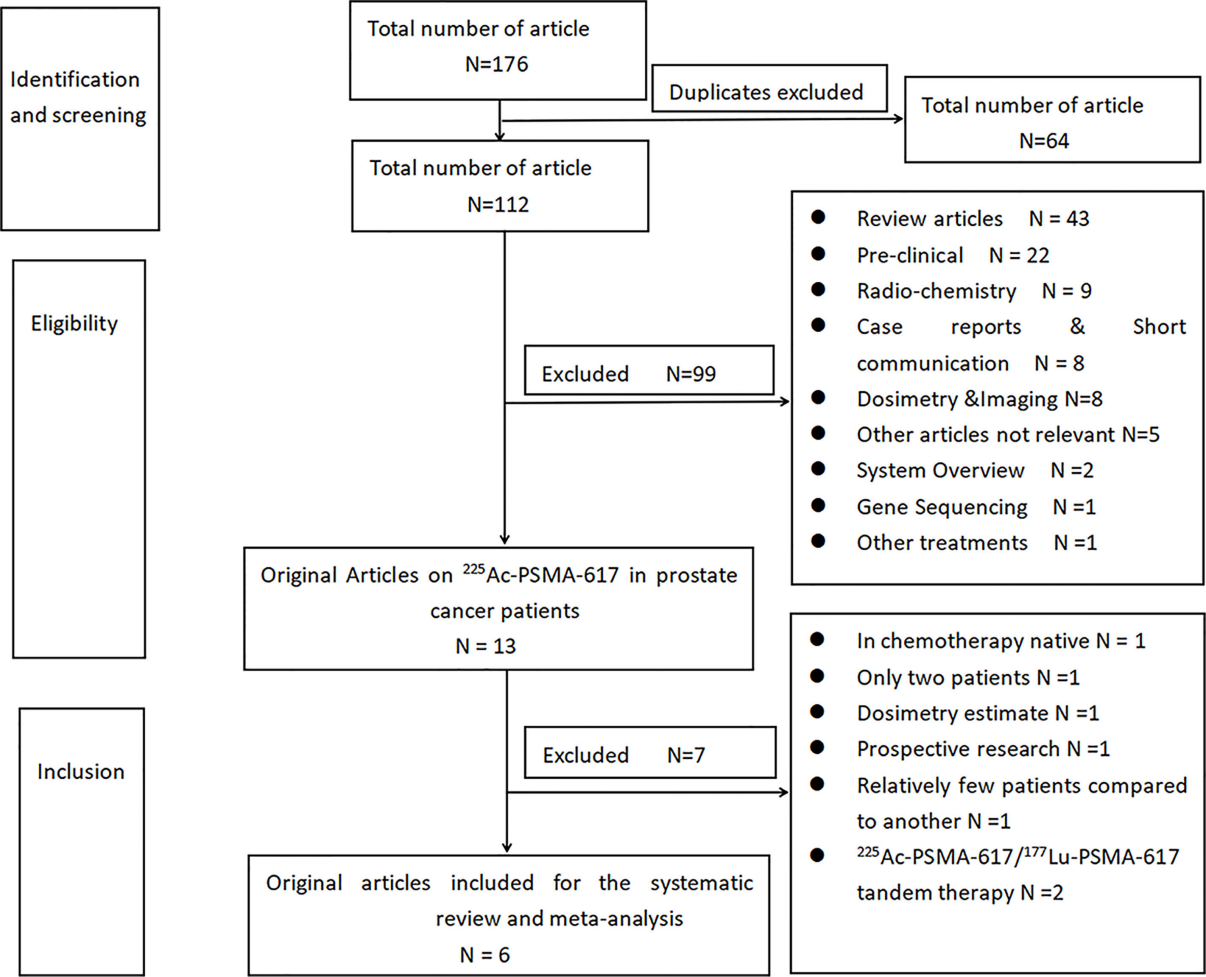
Funnel plot for >50% PSA decline after treatment.

The qualitative and quantitative evaluation of publication bias used funnel chart and Egger test (**Figures 4**, **5**). The results of any PSA decline indicated that there was no significant publication bias (P = 0.081). The Egger test result of greater than 50% PSA decline suggested that there was no significant publication bias (P = 0.105).

## Discussion

Currently, ^225^Ac-PSMA-617 targeted therapy for prostate cancer is undergoing trials in different countries. ^225^Ac has shown encouraging effect in the study of mCRPC patients, but since most of the trials are small samples and mostly retrospective, there are only few systematic reviews of ^225^Ac-PSMA-617 TAT. This meta-analysis evaluated the efficacy and safety of ^225^Ac-PSMA-617 TAT in mCRPC patients from retrospective studies published so far. The results showed that ^225^Ac targeted therapy for prostate cancer patients had a significant therapeutic effect and low toxicity. More than 80% of patients had any PSA decline, and more than 60% of patients had greater than 50% PSA decline. All patients who received this treatment had previously received second/third-line treatments such as abilaterol, enzalutamide, apalutamide or ^177^Lu-PSMA-617 and all failed. With ^225^Ac as a rescue treatment attempt, the results showed that OS and PFS were 12.5 months and 9.1 months. Approximately 54% of patients had complete or partial molecular reactions. After the failure of previous androgen receptor inhibitor (ARPI) treatment of prostate cancer, treatment with abiraterone or enzalutamide had an OS of 4 months, and cabazitaxel OS was 13–14 months. In contrast, the OS treated with ^177^Lu was 15.3 months (28). This study showed that the OS of ^225^Ac treatment of prostate cancer was better than the standard second-line/third-line treatment. Another study reported that giving ^177^Lu before docetaxel treatment produced a better PSA response than after docetaxel treatment (29). In a meta-analysis of randomized controlled trials in patients with mCRPC, the benefits and harms of eight third-line (L3) treatments for prostate cancer were evaluated. Compared with treatment with abiraterone, enzalutamide, mitoxantrone or cabazitaxel, PSMA PRLT resulted in a higher rate of PSA decline and a 1.1-fold increase in PFS (30). Although it was a preliminary study, it had shown the great potential of targeted radionuclide therapy. The decrease of PSA reflected the killing ability of cells during the treatment, and the progression-free survival reflected the comprehensive effects of cell killing and regeneration during the treatment cycle. The overall survival rate reflected the comprehensive effect of progression-free survival and treatment. The decline in PSA cannot predict OS and PFS. On the contrary, when PSA progressed, it indicated shorter OS and PFS (12, 24). These results had important implications for the extensive terminal stages of cancer, especially for patients with mCRPC. Among clinical relevant toxic reactions, xerostomia was the most common adverse reaction. More than 70% of patients had different degrees of xerostomia, but most were mild and transient. Significant treatment-related toxicities were only seen in a few patients. Grade III anemia, leukopenia, thrombocytopenia, and nephrotoxicity were only seen in 7.5, 4.5, 5.5, and 3.0% of the patients. In addition, toxicities such as nausea, fatigue, dysgeusia, indigestion, and constipation could be observed. Only 3 treatment-related deaths were reported in one article (15).

In the treatment of mCRPC, health-related quality of life is an important parameter to evaluate the subjective experience of the disease and its treatment. Most patients with mCRPC have bone metastases, which can lead to a significant incidence of bone pain and bone-related events. In addition, there will be a lot of general symptoms, such as fatigue, anorexia, bladder and intestinal disorders, nausea, vomiting, and sleep disturbances. Treatment-related adverse reactions may aggravate the deterioration of the quality of life of these patients. In this case, any new therapeutic drug not only needs to prove its survival benefit, but also needs to prove its impact on the quality of life of the patient. ^225^Ac-PSMA-617 treatment significantly improved health-related quality of life. Examples include physical symptoms such as pain, difficulty urinating, fatigue, and limited physical activity. In the van der Doelen, Feuerecker, and Sen studies, the European Organization for Cancer Research and Treatment (EORTC-QLQ30) quality of life questionnaire was used to evaluate patients (31). In the questionnaire assessment of van der Doelen and Sen, compared with baseline, pain was significantly improved, the use of analgesics was reduced, and the responses to analgesics were also improved. In addition, Sen et al. used the Standard Pain Numerical Scale (NPS) and Brief pain Inventory Questionnaire (BPI) for multidimensional pain assessment (32). Eight weeks after the second dose of ^225^Ac-PSMA-617 treatment, the NPS score dropped from baseline 5 points to 1 point. BPI measures the interference of pain on general activities, sleep, and mood, and had a significant improvement compared with baseline. The NCCN-FACT-FPSI-17 (version 2.0) (FACIT.org, Ponte Vedra, Florida, USA) questionnaire was used by Satapathy for evaluation (33), and the results showed that pain had also been significantly improved. For other aspects, van der Doelen showed greater improvement in fatigue and dyspnea; Satapathy showed significant improvement in dysuria, bone pain, fatigue and physical activity limitation; Feuerecker showed improvement in social function; Sen Showed significant improvement in fatigue, insomnia and constipation compared with baseline.

The PERCIST is only standardized for ^18^F-FDG PET/CT imaging. The complete and partial molecular responses observed on ^68^GA-PSMA PET/CT scans are still controversial. Therefore, it is challenging to accurately assess the treatment response of mCRPC patients. Velez et al. (34) showed that PERCIST 1.0 could provide important prognostic information for mCRPC patients receiving systemic chemotherapy, especially when combined with PSA treatment response criteria. More large-scale trials are needed to test the accuracy of ^68^GA-PSMA PET/CT in the evaluation of treatment response. In addition, the choice of treatment regimen and dosage is empirical. Most studies use 100 KBq/kg, and the treatment cycle ranges from 1 to 8 cycles. However, the effect of this targeted therapy is related to the expression level of PSMA. Although the expression of PSMA is closely related to hormone resistance and disease progression, the expression of PSMA in different metastases is heterogeneous. Moreover, the interaction between systemic therapy and PSMA expression has not been studied clearly (35), so the individualized treatment plan and dose selection for patients still need to be explored continuously. The current study inclusion criteria are all patients with positive PSMA expression, and the results showed a good treatment effect. However, for patients with lack or low expression of PSMA, whether these patients can still benefit from PSMA RLT, and how to choose a reasonable and effective combination treatment plan still needs continuous follow-up research.

This study also has certain limitations. All included studies were single-arm retrospective observational studies, the sample size of the trial was small, and the risk of bias was high. In addition, these trials were heterogeneous in terms of research design, other diseases, the course of prostate cancer, previous treatments, and the degree of PSMA expression. The follow-up time was short, and there were few studies on the comprehensive evaluation of molecular response and survival, which limited the accuracy of observation and evaluation of these indicators.

## Conclusion

^225^Ac-PSMA-617 is an effective and safe treatment option for mCRPC patients, and the treatment-related side effects caused by it are relatively low. However, 225Ac-PSMA-617 is in the clinical trial stage, and the efficacy and safety of its treatment plan still need to be evaluated in a high-quality, multi-center and prospective multi-arm randomized controlled trial.

## Data Availability Statement

The original contributions presented in the study are included in the article/supplementary material. Further inquiries can be directed to the corresponding author.

## Author Contributions

JM, LL, and CZ contributed to conception and design of the study. JM organized the database. JM, LL, and TL performed the statistical analysis. JM wrote the first draft of the manuscript. LL, TL, and WG wrote sections of the manuscript. All authors listed have made a substantial, direct, and intellectual contribution to the work and approved it for publication.

## Conflict of Interest

The authors declare that the research was conducted in the absence of any commercial or financial relationships that could be construed as a potential conflict of interest.

## Publisher’s Note

All claims expressed in this article are solely those of the authors and do not necessarily represent those of their affiliated organizations, or those of the publisher, the editors and the reviewers. Any product that may be evaluated in this article, or claim that may be made by its manufacturer, is not guaranteed or endorsed by the publisher.
